# Molecular Diagnostic Tools for Detection and Differentiation of Phytoplasmas Based on Chaperonin-60 Reveal Differences in Host Plant Infection Patterns

**DOI:** 10.1371/journal.pone.0116039

**Published:** 2014-12-31

**Authors:** Tim J. Dumonceaux, Margaret Green, Christine Hammond, Edel Perez, Chrystel Olivier

**Affiliations:** 1 Agriculture and Agri-Food Canada, Saskatoon Research Centre, Saskatoon, Saskatchewan, Canada; 2 Department of Veterinary Microbiology, University of Saskatchewan, Saskatoon, Saskatchewan, Canada; 3 Canadian Food Inspection Agency, Centre for Plant Health, Sidney, British Columbia, Canada; 4 Instituto de Biotecnologia y Ecologia Aplicada (INBIOTECA), Universidad Veracruzana, Avenida de Las Culturas Veracruzanas, Xalapa, Veracruz, Mexico; Naval Research Laboratory, United States of America

## Abstract

Phytoplasmas (‘*Candidatus* Phytoplasma’ spp.) are insect-vectored bacteria that infect a wide variety of plants, including many agriculturally important species. The infections can cause devastating yield losses by inducing morphological changes that dramatically alter inflorescence development. Detection of phytoplasma infection typically utilizes sequences located within the 16S–23S rRNA-encoding locus, and these sequences are necessary for strain identification by currently accepted standards for phytoplasma classification. However, these methods can generate PCR products >1400 bp that are less divergent in sequence than protein-encoding genes, limiting strain resolution in certain cases. We describe a method for accessing the chaperonin-60 (*cpn*60) gene sequence from a diverse array of ‘*Ca.*Phytoplasma’ spp. Two degenerate primer sets were designed based on the known sequence diversity of *cpn*60 from ‘*Ca.*Phytoplasma’ spp. and used to amplify *cpn*60 gene fragments from various reference samples and infected plant tissues. Forty three *cpn*60 sequences were thereby determined. The *cpn*60 PCR-gel electrophoresis method was highly sensitive compared to 16S-23S-targeted PCR-gel electrophoresis. The topology of a phylogenetic tree generated using *cpn*60 sequences was congruent with that reported for 16S rRNA-encoding genes. The *cpn*60 sequences were used to design a hybridization array using oligonucleotide-coupled fluorescent microspheres, providing rapid diagnosis and typing of phytoplasma infections. The oligonucleotide-coupled fluorescent microsphere assay revealed samples that were infected simultaneously with two subtypes of phytoplasma. These tools were applied to show that two host plants, *Brassica napus* and *Camelina sativa*, displayed different phytoplasma infection patterns.

## Introduction

‘*Candidatus* Phytoplasma’ spp. are obligate intracellular Mollicutes that live and reproduce in the phloem tissue of plants and are transmitted by phloem-feeding leafhoppers, planthoppers, and psyllids [1]. Phytoplasmas infect a wide range of plants, including many species that are agriculturally significant [2]. These microorganisms induce developmental changes in infected plants, from virescence and phyllody to stunting and decline. In agronomically important crops, phytoplasma infection can alter inflorescence morphology and dramatically decrease seed set and/or fruit quality [3].

‘*Ca.*Phytoplasma’ spp. are generally difficult to culture *in vitro* and a recent report claiming to have cultured these bacteria [4] has been met with skepticism [5]. Diagnosis and differentiation of phytoplasma infection has therefore relied on molecular methods, principally PCR targeting a wide variety of regions within the 16S–23S rRNA genes [6]. The use of observed or virtual RFLP fingerprinting and DNA sequencing methods to differentiate ‘*Ca.*Phytoplasma’ spp. allowed the grouping of phytoplasma strains that can be divergent in 16S rRNA-encoding sequences [3]. 16S–23S rRNA gene sequences have been effectively used to differentiate and classify phytoplasma strains [7]–[10], resulting in the identification of at least thirty groups of phytoplasma. Molecular diagnostic methods targeting 16S rRNA-encoding genes, including conventional PCR combined with direct sequencing and quantitative PCR (qPCR), have been employed to detect phytoplasma infection in plants and insects [6], [11], [12]. Limitations of rRNA-encoding gene targeted typing methods include the length of the amplicon generated (which can be>1500 bp) as well as the inability of ribosomal RNA-encoding gene sequences to differentiate certain subgroups of phytoplasma. [13]–[17].

These limitations have motivated the search for other molecular markers for the detection of phytoplasma infections. Protein-encoding genes are known to provide increased strain resolution compared to rRNA-encoding genes [18], but the use of protein-encoding genes requires tools such as PCR primers that are able to access the genes from genomes that may be highly divergent in sequence. The gene encoding the 60 kDa chaperonin (*cpn*60) has been shown to meet the criteria set out by the International Barcode of Life consortium for a barcode for the domain Bacteria [19]. Indeed it has been shown that *cpn*60 sequences (often called *groEL* or *hsp*60) provide superior strain resolution for ‘*Ca.*Phytoplasma’ spp. compared to rRNA-encoding gene targets [14], [15]. However, the PCR amplification methods described for accessing *cpn*60 sequences from unknown and potentially divergent phytoplasma genomes [15] have been limited to specific subgroups (e.g. 16SrI, or ‘*Ca.*P. *asteris*’) and result in the generation of a sequence of ∼1.4 kb, which is inconvenient for the rapid generation of novel sequences by direct sequencing of PCR products.

The *cpn*60 universal target (*cpn*60 UT) is a 549–567 bp (183–189 amino acid) region of the gene corresponding to nucleotides 247–828 of *E. coli groEL* [20]. The *cpn*60 UT can be amplified from nearly all bacteria using a set of degenerate universal primers [21], [22], and *cpn*60 UT sequences have been used to detect, identify, and differentiate a wide variety of bacterial groups [23]–[27]. Furthermore, the *cpn*60 UT has proven to be a useful target for the development of molecular diagnostic assays such as qPCR [28] and hybridization-based assays [24], [29] for other non-phytoplasma bacteria as well as specifically used to detect phytoplasma species using a loop-mediated isothermal DNA amplification assay [30].

The potential utility of previously described tools for accessing *cpn*60 is limited for ‘*Ca.*Phytoplasma’ spp. as samples will contain non-target bacterial and eukaryotic DNA, decreasing the specificity of the *cpn*60-based molecular diagnostic. However, the availability of full-length *cpn*60 gene sequences for certain ‘*Ca.*Phytoplasma’ spp., combined with the low G/C content observed in all ‘*Ca.*Phytoplasma’ spp. (approximately 21–28% G/C in 11 reported genomes), led us to investigate the possibility of designing a novel genus-level specific PCR-based assay for generating *cpn*60 UT sequences for additional ‘*Ca.*Phytoplasma’ spp. Our objective in the present study was to determine the feasibility of exploiting the *cpn*60 UT as a molecular diagnostic target for the detection and differentiation of ‘*Ca.*Phytoplasma’ spp. We describe two sets of PCR primers that successfully amplify the *cpn*60 UT from a wide variety of ‘*Ca.*Phytoplasma’ spp. that are highly divergent in sequence (61–98% identity). We used these sequences to develop molecular diagnostic assays that demonstrated differences in host plant infections between two related oilseed crops, *Brassica napus* and *Camelina sativa*.

## Materials and Methods

### Provenance of plant samples and DNA extracts

In 2012, plants belonging to the species *Linum usitatissimum*, *Brasica napus*, *Brassica napobrassica*, *Allium cepa*, *Daucus carota* and *Thlapsi arvense* that showed severe Aster yellows (AY) symptoms were sampled from fields located near Saskatoon, Saskatchewan, Canada (52.13° N, 106.68° W). DNA extracts of ‘*Ca.*P. *phoenicium*’ originated from infected plant tissue of *Catharanthus roseus* located in near Madruga, Mayabeque, Cuba (22.91°N, 81.85°W). These plants were sampled from areas that did not require specific permissions, and no endangered or protected plant species were sampled for this work. DNA extracts of Stolbur, Bois noir, (16SrXII) originated from 16SrXII-infected *C. roseus* sent by Dr. M. Maixner (JKI-Institute for Plant Protection in Fruit Crops and Viticulture, Germany), DNA extracts of Aster yellows (16SrI and 16SrI-C) and DNA extracts of apple proliferation group or European stone fruit yellows and Pear decline (16SrX-B and 16SrX-C) were obtained from Dr. A. Bertaccini (University of Bologna, Italy) and Dr. X. Foissac (INRA-Bordeaux, France), respectively. Plants of *Ligustrum sinense* and *C. roseus*, infected with AY-16SrI-A were maintained in the laboratory and used as positive controls. Healthy *C. roseus* and *B. napus* grown in an insect-free growth chamber were used as negative controls. A comprehensive list of all source data obtained for strains of ‘*Ca.*Phytoplasma’ spp. analyzed in this study is shown in S1 Table.

### Plant sampling and DNA extraction

Approximately 0.1 g of finely cut leaf and stem tissues was placed in a well of a 96-well plate and lyophilized for 48 hours (−40°C, 0.120 mBar). Tissue was homogenized using a TissueLyser II (Qiagen) with one-3 mm glass bead at 30 Hz for 2 min. DNA was extracted using a modified hexadecyltrimethylammonium bromide (CTAB)-based method [31]. Briefly, homogenized tissue was suspended in 250 µL of a solution of 55 mM CTAB, 100 mM Tris-Cl pH 8.0, 20 mM EDTA, and 1.4 M NaCl. 2-mercaptoethanol was added to this solution (4 µL/mL) immediately prior to DNA extraction and the samples were vortexed briefly and incubated at 65°C for 1 hour. Samples were extracted with 1.0 volume of chloroform, centrifuged, then the upper phase was transferred to a fresh tube and 1.0 volume of isopropanol was added. Samples were centrifuged, dried, and the DNA pellets were dissolved in 200 µL of TE (10 mM Tris-Cl pH 8.0, 1 mM EDTA) containing RNase A (0.2 mg/mL). DNA extracts were stored at −20°C.

### Design of oligonucleotide-based tools targeting *cpn*60 of ‘*Ca.*Phytoplasma’ spp

Sequences for all amplification primers and hybridization probes along with optimized amplification conditions are shown in S2 Table. Primers for phytoplasma PCR amplification were based on the *cpn*60 UT primer annealing sites [22], but were adapted to phytoplasma sequences using full-length *cpn*60 genes from public databases (www.cpndb.ca and www.ncbi.nlm.nih.gov). One set of primers (H279p/H280p) was based on 18 full-length *cpn*60 sequences, which primarily represented the AY group. A second set of PCR primers (D0317/D0318) was designed based on the full-length *cpn*60 reported in the Peanut Witches’ Broom (PnWB) Phytoplasma genome sequence reported by Chung *et al*. [32]. Hybridization probes for the fluorescent microsphere detection assay were designed using PrimerPlex v2.62 (Premier Biosoft, Palo Alto, CA, USA).

### PCR amplification and sequencing

A dilution series of each DNA extract (neat, 1∶2, 1∶5, 1∶10, 1∶20, 1∶50, and 1∶100) was routinely analyzed to compensate for the possibility of PCR inhibition. Samples were analyzed using 16S rRNA gene-targeted universal bacterial PCR primers [33] to ensure that amplifiable bacterial DNA was present in the extract at the dilutions analyzed. *cpn*60 UT amplicons were generated under the following conditions: 1×PCR buffer (Life Technologies); 2.5 mM MgCl_2_; 0.4 µM each primer (S1 Table); 0.5 mM each dNTP; and 1 U Taq DNA polymerase (Life Technologies). PCR primers were synthesized by IDT (Coralville, IA). PCR cycling conditions for *cpn*60 were 95°C, 3 min (1x) followed by 30 cycles of 95°C, 30 sec; 42°C, 30 sec; 72°C, 30 sec. A final extension at 72°C (5 min) was performed. PCR products were either directly sequenced using the amplification primers, or were first ligated into the vector pGEM-T Easy (Promega, Madison, WI USA) according to the manufacturer’s recommendations, transformed into chemically competent *E. coli* JM109 (Promega), and sequenced using plasmid-targeted primers T7/SP6. For amplification of 16S–23S rRNA-encoding loci, primers P1 and Tint [6] were used with the recommended amplification conditions.

### Sensitivity, specificity, and limit of detection of *cpn60*-based molecular diagnostic assays

The diagnostic sensitivity and specificity, along with 95% confidence intervals, were calculated using 192 DNA extracts from field-collected plant tissues according to standard methods [34]. The results of 16S–23S rRNA locus-targeted PCR amplified with P1/Tint [6] were used as the gold standard to call positive and negative samples. The limit of detection (LOD) of each assay was determined by probit analysis of 8 replicates each of a dilution series of non-linearized plasmid DNA containing 10^7^–10^1^ copies/reaction. Plasmids were diluted in 10 mM Tris-Cl, pH 8.0 containing 5 ng/ml yeast tRNA and stored in DNA low-bind tubes (Eppendorf). Probit analysis was conducted using SPSS (IBM Corp. Released 2010. IBM SPSS Statistics for Windows, Version 21.0) and the LOD was specified as the copy number that was predicted to yield a positive result in 95% of assays performed, as specified by Bustin et al. [35]. Plasmid templates for the LOD assays were as follows: for H279p/H280p, AY-OY-M; for D0317/D0318, FD; for P1/Tint, PCR product from infected flax plants cloned into pGEM-T Easy.

### Phylogenetic analysis of phytoplasma *cpn*60 sequences

Sequences were aligned using ClustalW [36] and analyzed using the Maximum Likelihood method based on the Tamura-Nei model [37]. Trees were bootstrapped 1000 times. Analysis was conducted in MEGA5 [38].

### Oligonucleotide-coupled fluorescent microsphere diagnostic assay

Phytoplasma *cpn*60 was amplified using the phytoplasma PCR primer sets with the upstream primers modified with biotin and four phosphorothioate-modified bases (S2 Table). Amplicons were rendered single-stranded by digestion with T7 exonuclease and hybridized to oligonucleotide-coupled fluorescent microspheres (Bio-Rad) as described previously [29], [39]. Hybridization probes had a 5-amino C12 modification at the 5′ end to facilitate bead coupling (IDT, Coralville, IA). The results were analyzed using a Bio-Plex instrument (Bio-Rad) and Bio-plex Manager software (v6.1.0.727). Duplicate aliquots of each amplification product were hybridized to the fluorescent microspheres, and in some cases duplicate amplification reactions were analyzed, for a total of 2 or 4 hybridization assays per sample. The median fluorescence intensity (MFI) of each bead in every sample was determined by measuring the fluorescence of 100 microspheres. The MFI of each microsphere in PCR-amplified samples was compared to that of a negative control (no template) using a Student’s t-test (one-tailed distribution). Positive results were scored as those with an MFI>120 that were significantly greater than the negative control at *P*<0.01.

### Determination of phytoplasma strains infecting oilseed crops at a single location

Breeding lines and commercially available cultivars of *Camelina sativa* and *Brassica napus* plants were grown at the Agriculture and Agri-Food Canada research farm at Saskatoon, Saskatchewan, Canada, during the growing season of 2012. Plants were seeded in two rows in plots 3.0 m in length with 0.30 m between rows and 100 seeds per row, using a randomized complete block design. Rows of barley were used at the edge of each bloc. Tissue was harvested from plants showing signs of phytoplasma infection and DNA was extracted as described above. DNA was used as a template for nested PCR using primers P1/P6 [40] and R16F2/R16R2 [41] targeting the 16S–23S rRNA-encoding locus and products were directly sequenced using R16F2/R16R2. The same extracts were used as template for *cpn*60-targeted PCR using primers H279p/H280p and products were either directly sequenced using the amplification primers or were subjected to the fluorescent microsphere hybridization assay as described above.

## Results

### Design of oligonucleotide-based tools targeting *cpn60* of ‘*Ca.*Phytoplasma’ spp

Initial experiments aimed at developing a *cpn*60-targeted phytoplasma PCR assay used universal phytoplasma primer AY-groEL-F [15] and primer H280p (S2 Table). This primer set generated a PCR product of 826 bp that contained the entire *cpn*60 UT along with 275 bp of upstream sequence. PCR products were generated using this primer set from DNA extracted from infected tissues of *Linum usitatissimum*. However, analysis of DNA extracted from infected *Brassica napus* plants revealed that this primer set had a very low sensitivity compared to P1/Tint (0.489 with 90 positives analyzed), so a new primer was designed based on the sequence of the *cpn*60 UT universal primer annealing site.

**Figure 1:**
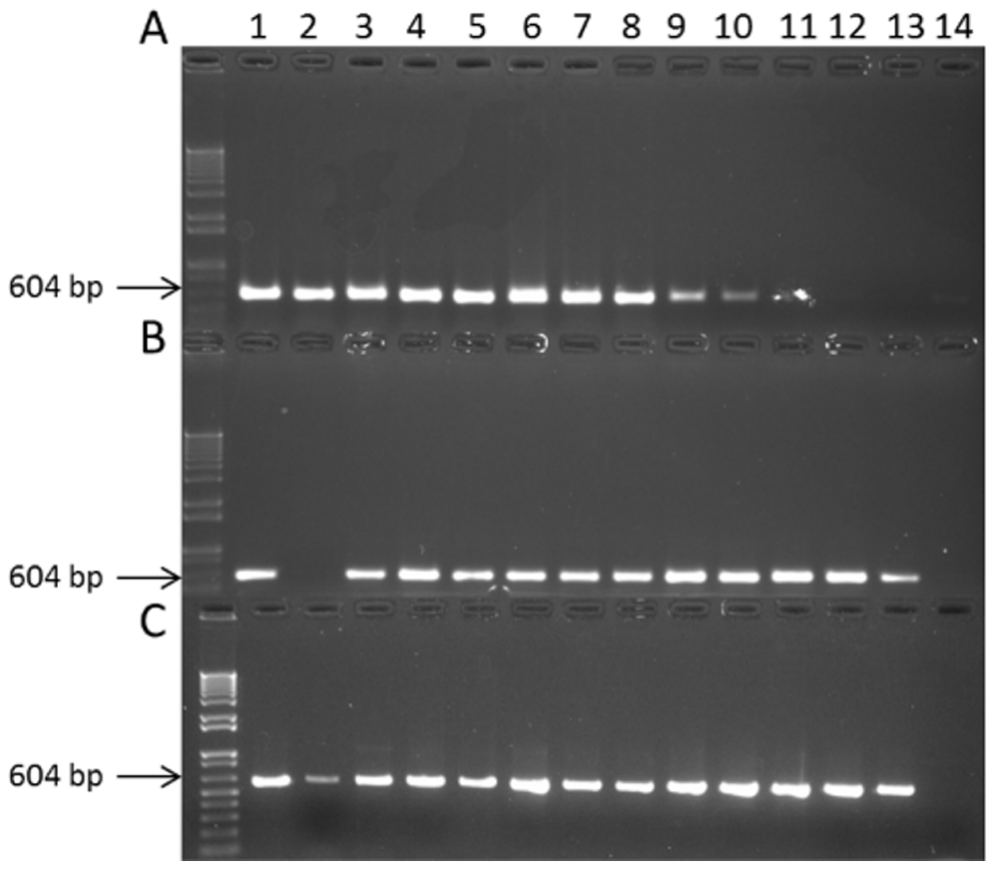
Breadth of detection of the *cpn*60-targeted PCR assays for ‘*Ca.*Phytoplasma’ spp. **A.** Samples amplified using primer set H279p/H280p. **B.** Samples amplified using primer set D0317/D0318. **C.** Samples amplified using an optimized cocktail consisting of a 1∶7 molar ratio of primer sets H279p/H280p:D0317/D0318. For all panels: Lane 1, Apple proliferation (‘*Ca.*P. *mali*’); lane 2, Peach yellow leaf roll (‘*Ca.*P. *pyri*’); lane 3, European stone fruit yellows (‘*Ca.P. prunorum*’); lane 4, Bois noir – isolate Pyrenées Orientalis (‘*Ca.*P. *solani*’); lane 5, AY strain OY-M (‘*Ca.*P. *asteris*’); lane 6, AY strain COL (‘*Ca.*P. *asteris*’); lane 7, AY strain CVB (‘*Ca.*P. *asteris*’); lane 8, AY strain AY-WB (‘*Ca.*P. *asteris*’); lane 9, Brazilian huanglongbing phytoplasma (‘*Ca.*P. *phoenicium*’); lane 10, Flavescence dorée (‘*Ca.*P. *ulmi*’); lane 11, Bois noir – isolate VL-06-1-20, Lebanon (‘*Ca.*P. *solani*’); lane 12, Rubus stunt (‘*Ca.*P. *ulmi*’); lane 13, Ash yellows (‘*Ca.*P. *fraxini*’); lane 14, no template control.

This primer set, H279p/H280p (S2 Table), contained degenerate bases to capture the breadth of sequence heterogeneity observed in 18 full-length Phytoplasma *cpn60* genes found in the cpnDB (www.cpnDB.ca). The sequences available for primer design were principally derived from the AY group, along with ‘*Ca.*P. *mali*’ (Apple Proliferation Phytoplasma). Selectivity of this primer set for ‘*Ca.*Phytoplasma’ spp. was increased by using a low T_a_ (42°C), exploiting the low G/C content observed in phytoplasma genes [14], [15], [32]. Primer set H279p/H280p successfully generated amplicons from a wide array of DNA extracted from infected plant tissues, including strains corresponding to ‘*Ca.* P. *asteris*’, ‘*Ca.* P. *solani*’, ‘*Ca.* P. *mali*’, ‘*Ca.* P. *prunorum*’, and ‘*Ca.* P. *pyri*’ (Fig. 1A). However, this primer set failed to amplify product from other ‘*Ca.* Phytoplasma’ species.

**Figure 2:**
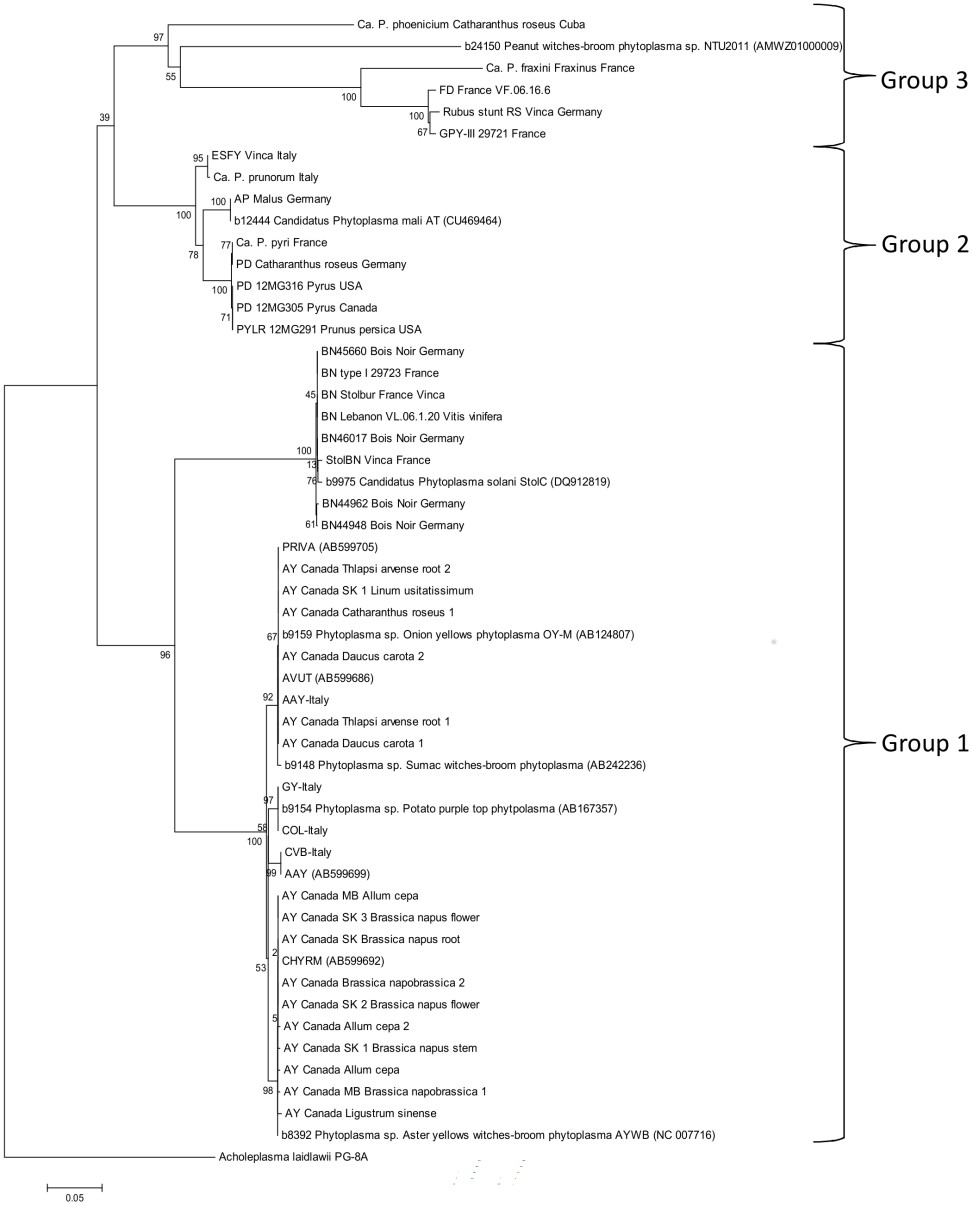
Molecular phylogeny (maximum likelihood) of ‘*Ca.*Phytoplasma’ spp. based on the sequences of the *cpn*60 UT. Reference strains are indicated by public database accession numbers (cpnDB id before strain name and GenBank accession numbers in parentheses). Numbers next to the nodes indicate bootstrap support based on 1000 replicates. The *cpn*60 UT sequence obtained from the genome of *Acholeplasma laidlawii* strain PG-8A (GenBank accession no NC_010163.1) is included as the outgroup. See S1 Table for a list of strain abbreviations used on this tree. Groupings correspond to those suggested by Chung et al. [32].

The availability of the complete genome sequence for Peanut Witches’ Broom Phytoplasma (PnWB; 16SrII group) [32] revealed the extreme sequence diversity to be expected at this locus: PnWB *cpn*60 was only 63–65% identical to any other phytoplasma *cpn*60 sequence reported, and would not have been expected to amplify with H279p/H280p. Therefore, a second set of primers was required to capture the diversity of phytoplasma *cpn*60 sequences. Primer set D0317/D0318 successfully generated *cpn*60 UT amplicon from ‘*Ca.* Phytoplasma’ spp., including ‘*Ca.*P. *phoenicium*’, ‘*Ca.*P. *fraxini*’, and ‘*Ca.* P. *ulmi*’. These templates did not amplify with H279p/H280p (Fig. 1B) and were highly divergent in *cpn*60 UT sequence compared to the other samples (Fig. 2). Based on these results, various ratios of H279p/H280p and D0317/D0318 were tested to determine if all of the observed sequence diversity of phytoplasma *cpn*60 could be captured in a single PCR primer cocktail. A ratio of 1∶7 H279p/H280p:D0317/D0318 successfully generated *cpn*60 amplicon from all of the ‘*Ca.*Phytoplasma’ spp. tested in a single reaction (Fig. 1C).

In general, primer set H279p/H280p amplified *cpn*60 genes from ‘*Ca.*P. *solani*’ (Bois noir), ‘*Ca.*P. *asteris*’, and the 16SrX group and was not effective on other samples (Fig. 1). The only exception to this was ‘*Ca.*P. *solani*’ (Bois noir) isolate VL-06-1-20, Lebanon, which repeatedly did not amplify with primer set H279p/H280p (Fig. 1A, lane 11), yet had a sequence that was identical to those generated for other strains of ‘*Ca.*P. *solani*’ (Fig. 2), all of which amplified with that primer set. However, this sample was amplifiable with primer set D0317/D0318 as well as the optimized cocktail (Fig. 1 B, C-lane 11).

### Diagnostic utility of *cpn60*-targeted amplification compared to 16S-23S-targeted PCR assay

Using 192 DNA extracts from infected and uninfected *B. napus* as template, the sensitivity of the *cpn*60-targeted phytoplasma PCR (H279p/H280p) was high when compared to the 16S-23S-targeted PCR (P1/Tint), with 93% of 16S-23S-positive samples also testing positive with the *cpn*60-targeted assay (Table 1). However, the specificity observed was low: only 44% of negative samples tested negative using the *cpn*60 assay. This suggested that the *cpn*60 assay yielded a high rate of false positives, which may be expected due to the fact that the universal primer hybridization sites were used for this assay. We therefore determined the *cpn*60 amplicon DNA sequences from those 39 discordant samples that tested negative by 16S–23S PCR but positive by *cpn*60-targeted PCR. The DNA sequences obtained from 38 of the 39 samples (1 reaction failed) were identical or nearly identical to those of ‘*Ca.* P. *asteris*’ (16SrI group): AY-WB (GenBank NC_007716.1) and AY-OY-M (GenBank AB124807) (Fig. 2). This confirms that those DNA extracts, which had originally tested negative for phytoplasma using the 16S–23S assay, were indeed positive. The LOD of the *cpn60*-targeted assays was examined using serial dilutions of plasmid DNA (Table 2). Probit analysis revealed that the LOD of the *cpn*60-targeted assays was ∼10^3^ copies, which was about 10-fold lower than that observed for the 16S rRNA gene-targeted P1/Tint assay (Table 2).

**Table 1:** Sensitivity and specificity of *cpn*60-targeted PCR assay compared to 16S–23S PCR.

	P1/Tint PCR results		
*cpn*60 PCR results^a^	positive	negative	Total
positive	114	39	153
negative	9	30	39
total	123	69	192
		95% CI^b^	
*cpn*60 PCR sensitivity	0.927	0.046	
*cpn*60 PCR specificity	0.435	0.117	

Positives and negatives were defined by the results of the P1/Tint assay and the corresponding numbers of positive and negative samples identified by the *cpn60*-targeted assay are indicated [34]. The sensitivity of the *cpn60* PCR using P1/Tint as a gold standard was 0.927 (114 positives of 123), with a 95% confidence interval of 0.046. The specificity of the *cpn60* PCR using P1/Tint as a gold standard was 0.435 (30 negatives of 69), with a 95% confidence interval of 0.117.

a assayed using primer set H279p/H280p.

b CI, confidence interval.

**Table 2:** Limit of detection (LOD) of PCR assays.

gene target	primer set^a^	LOD (copy no. per assay)
*cpn*60	H279p/H280p	2355
*cpn*60	D0317/D0318	4952
16S–23S	P1/Tint	34626

a see S1 Table.

### Molecular phylogeny of ‘*Ca.*Phytoplasma’ spp. based on *cpn60* UT sequences

Sequences were determined for all of the phytoplasma *cpn60* UT amplicons generated in this study. Overall, the sequence diversity at the *cpn60* UT was high, with the maximum divergence (61% sequence identity) observed between ‘*Ca.*P. *fraxini*’ (Ash Yellows) and ‘*Ca.*P. *solani*’ (Bois noir). The most similar *cpn60* sequences were the sequences belonging to ‘*Ca.*P. *asteris*’ (AY), with 97–98% sequence identity observed. Phylogenetic analysis of all of the phytoplasma *cpn60* UT sequences generated in this study, along with selected reference strains from cpnDB, is shown in Fig. 2. A total of 12 distinct groupings could be discerned, including 4 closely related groups of identical sequences (AY-OY-M; COL; CVB; and AY-WB). These sequences shared 97–98% identity and the reference sequences were all identified as’*Ca.*P. *asteris*’ (16SrI). Most of the infected plant samples we obtained from Canada yielded *cpn60* sequences that clustered into these ‘*Ca.*P. *asteris*’-like (16SrI) groups.

### Fluorescent microsphere assay for detection and identification of ‘*Ca.*Phytoplasma’ spp

**Figure 3:**
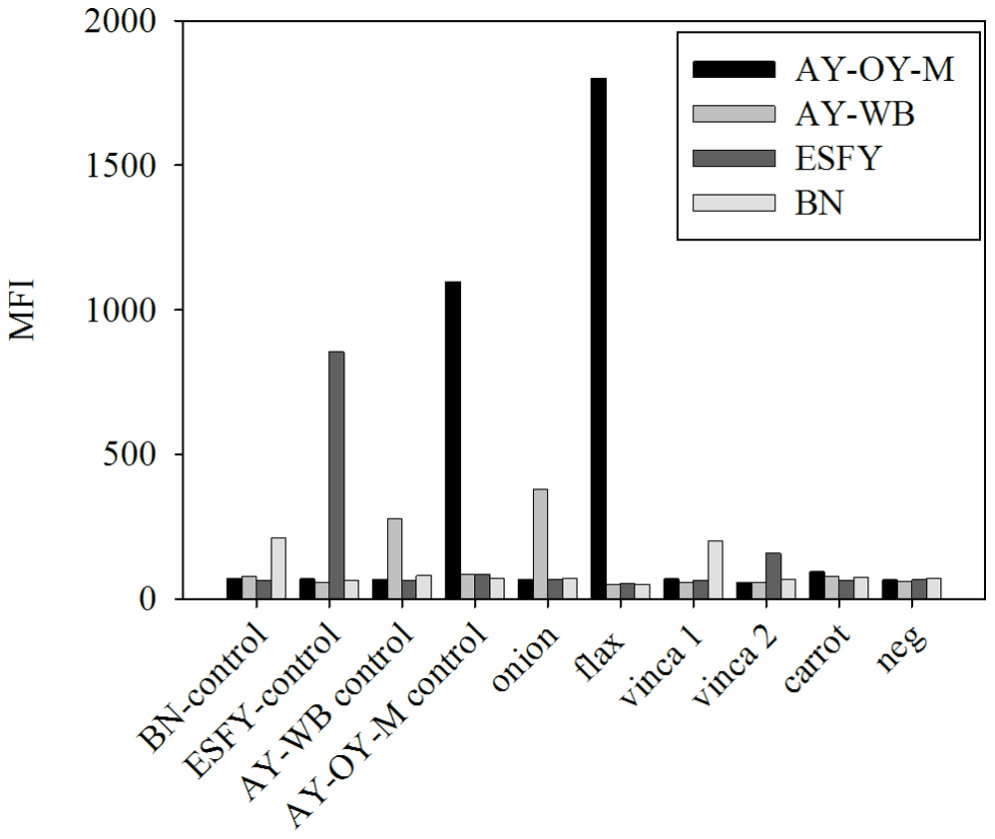
*cpn*60-targeted fluorescent microsphere hybridization assay to detect ‘*Ca.*Phytoplasma’ spp. Results are shown for a 4-plex assay (format used for analysis of 192 *B. napus* DNA extracts) on plasmid DNA controls (10^7^ copies/PCR) and on genomic DNA extracted from various infected plant tissues. Beads with a positive hybridization signal in each sample are identified (*). Samples from infected plant tissues are those described in S1 Table (onion, item #25; flax, item #41; vinca1, item #33; vinca2, item #35; carrot, item #15). Abbreviations: MFI, median fluorescence intensity; BN, Bois Noir; ESFY, European Stone Fruit Yellows; AY, Aster Yellows.

**Figure 4:**
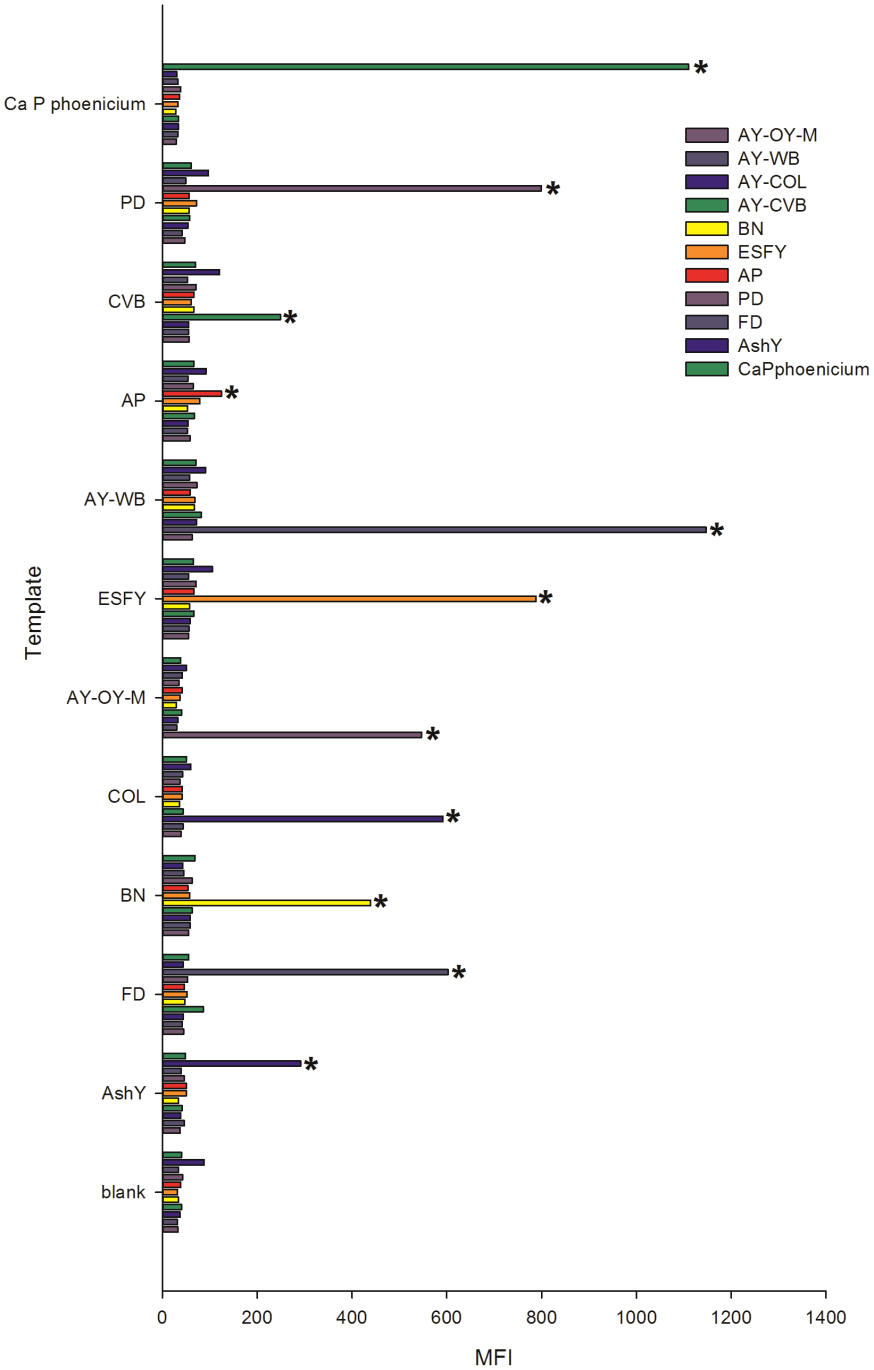
Expanded 11-plex fluorescent microsphere hybridization assay. All templates were plasmid DNA controls (10^8^ copies/2 µl). Probe identities are shown in the legend. Beads with a positive hybridization signal in each sample are identified (*). Abbreviations: PD, Pear decline; AY, Aster yellows; AP, Apple proliferation; ESFY, European stone fruit yellows; BN, Bois noir, FD, Flavescence dorée; AshY, Ash yellows. CVB and COL are strains of Aster Yellows (S1 Table).

The *cpn*60 UT sequences generated using these primer sets were used to design a suite of hybridization probes for various ‘*Ca.*Phytoplasma’ spp. spanning the complete *cpn*60 UT sequence diversity that has been observed to date (S2 Table). The single exception to this was PnWB, for which a *cpn60* sequence, but not template DNA, was available. Probes were coupled to fluorescent microspheres, mixed together, and hybridized to single-stranded PCR products generated using the *cpn60*-targeted phytoplasma primer sets (S2 Table). Results generated from a 4-plex bead mixture are shown in Fig. 3. The signal intensity generated using the plasmid DNA controls was highly variable, even though each contained the same copy number in the initial PCR (10^8^ copies). Since hybridization signal intensity can affect the definition of a positive result, we implemented a statistical definition of a positive result by specifying that each bead must generate a signal that is significantly greater than the background at *p*<0.01 using a Student’s t-test. Using this definition, we identified samples that were positive for various ‘*Ca.*Phytoplasma’ spp. from infected plant tissues, including AY-OY-M, AY-WB, ESFY, and Bois noir (Fig. 3). All of the 11 hybridization probes generated a signal only with the intended target (Fig. 4). Samples containing DNA from ‘*Ca.* P. *asteris*’ isolates AY-OY-M, AY-CVB, AY-COL, and AY-WB were readily distinguishable despite the fact that the *cpn*60 UT sequences of these strains were 97–98% identical (Figs. 3, 4).

The fluorescent microsphere assay (4-plex format) was implemented to examine 192 DNA extracts from *B. napus*. A summary comparing the results of *cpn*60 and 16S–23S PCR is shown in Table 3, and the complete results of all assays are shown in S3 Table. The fluorescent microsphere assay employed a PCR step that did not require agarose gel electrophoresis to determine a positive result and also returned strain-level information without the need for DNA sequencing. Overall the sensitivity of the PCR-fluorescent microsphere method was low, consistent with results we have observed with this assay using other samples (data not shown). However, the strain-level information returned for positive samples allowed the immediate determination of the strain of phytoplasma observed in each sample. In 29 of 30 cases in which both PCR product sequences and fluorescent microsphere hybridization results were obtained from the same templates, the results agreed. One sample (BN28T-94) appeared to be most closely related to AY-WB by direct sequencing of the PCR product but was positive for both AY-WB and AY-OY-M by the hybridization assay (S3 Table). Close examination of the sequence revealed that both sequences were present in the PCR product generated from this sample (S2 Fig. and S4 Table). This was subsequently confirmed by cloning this PCR product and sequencing several clones (data not shown). Similar double positive infections were noted in three other samples of *B. napus* (S3 Table).

**Table 3:** Summary of diagnostic assays conducted on 192 field-collected samples of *B. napus* from the 2012 growing season.

			samples with typing results			
test	total pos	total neg	AY-OY-M	AY-WB	AY-OM and AY-WB	Total^b^
*cpn*60 PCR-gel electrophoresis^a^	153	39	27	11	0	38
*cpn*60 PCR-fluorescent microsphere assay^a^	125	67	53	68	4	125
16S–23S PCR-gel electrophoresis	123	69	ND^c^	ND	ND	ND

Complete results for each sample are shown in S3 Table.

a assayed using H279p/H280p.

b only PCR products from samples that gave discordant results between P1/Tint (neg) and H279p/H280p (pos) were directly sequenced. One reaction failed so no typing information is available. For the fluorescent microsphere assays, all positive samples returned a typing result.

c ND, not determined.

### Comparison of phytoplasma infection patterns in host plants

The phytoplasma strains infecting two oilseed crops, *Camelina sativa* and *Brassica napus*, grown at the same geographic location at the same time were compared using 16S-23S rRNA and *cpn*60-encoding gene sequences. Within the infected *B. napus* plants examined, a mixture of two AY subtypes were identified using *cpn*60, with both AY-OY-M (65%) and AY-WB (35%) found in these samples (Table 4 and S5 Table). In contrast, only AY-OY-M was found in the *C. sativa* plants grown in the same location in the same year. The *cpn*60 UT sequences of these strains were 98% identical (S3 Fig.); however the 16S–23S rRNA-encoding sequences of the phytoplasma strains infecting the two oilseed crops were indistinguishable (S4 Fig.).

**Table 4:** Determination of *Phytoplasma* subtypes from host plants grown in 2012 at a single location in Saskatoon, SK, Canada (52.13° N, 106.68° W).

	Number of samples identified as AY subtype		
host	AY-OY-M	AY-WB	total
*B. napus*	42	23	65
*C. sativa*	70	0	70

## Discussion

Rapid, accurate diagnosis of phytoplasma infection and strain differentiation in host plants and insect vectors is essential to support disease surveillance, international trade in agricultural goods, and timing of interventions such as pesticide application to mitigate disease spread. Since ‘*Ca.*Phytoplasma’ spp. are obligate intracellular pathogens that are difficult or currently impossible to grow in culture, strain detection and differentiation has relied on methods based on the amplification of specific gene targets in the phytoplasma genome. Many different PCR methods have been developed targeting the 16S–23S rRNA-encoding gene region that can amplify targets from all known ‘*Ca.*Phytoplasma’ spp. [6], [40], and the phylogeny and classification of ‘*Ca.*Phytoplasma’ spp. has been described based on the sequences of this genomic region [7], [32].

While the 16S–23S rRNA-encoding region has proven to be very useful for the detection and differentiation of ‘*Ca.*Phytoplasma’ spp., certain disadvantages are associated with its use for routine screening. These limitations are highlighted by ongoing efforts to define alternative, protein-encoding molecular barcoding targets for phytoplasma detection and differentiation, which have resulted in an array of useful molecular tools becoming available for phytoplasma detection and differentiation, each with its own advantages and disadvantages. One such target is the elongation factor TU (*tuf* gene), which has been used to differentiate effectively strains of ‘*Ca.*P. *asteris*’ [42], and has been proposed as a useful molecular barcode for ‘*Ca.*Phytoplasma’ spp. [43]. Alternatively, a series of nine primer sets has been described that generate PCR products from a protein translocase subunit (*secY*) gene of a wide array of ‘*Ca.*Phytoplasma’ spp. [16]. The gene encoding RNA polymerase β-subunit (*rpoB*) has also been recently exploited as a molecular marker for phytoplasma [44]. All of these sequences are useful for differentiation of ‘*Ca.*Phytoplasma’ spp., and additional, non-ribosomal RNA-encoding molecular markers will provide further tools for the detection and differentiation of phytoplasma in plant and insect tissues.

Among the molecular markers proposed for DNA barcoding of phytoplasmas, only the gene encoding Cpn60 has been shown to be a molecular barcode for Bacteria that meets the criteria set forth by the International Barcode of Life in terms of barcode gap [19]. Furthermore, *cpn60* has been shown to provide high resolution differentiation of ‘*Ca*. P. *asteris*’ [14], [15]. However, the *cpn*60-targeted PCR assays that have been described are limited to certain subgroups, possibly due to the very high sequence divergence observed at this locus. The *cpn*60 UT has been exploited as a region of *cpn*60 that is accessible from nearly any bacterial genome (and many eukaryotic genomes) using a set of degenerate universal primers [22], and we sought to use this region as a basis for developing a PCR assay that can detect and differentiate ‘*Ca.*Phytoplasma’ spp. in plant- or insect-derived DNA extracts. The primer sets that we have described yielded a PCR product with a size that is amenable to single-pass Sanger sequencing (552–555 bp when trimmed of primer sequences) and that possessed a very wide range of sequence diversity (61–98% identity). These primers were used to amplify the *cpn*60 UT from 43 phytoplasma-infected samples representing seven widely divergent 16Sr groupings. PCR based on *cpn*60 was more sensitive than 16S-23S-based PCR and had a lower LOD (Table 2).

One potential limitation of the use of the *cpn*60 UT for phytoplasma detection is the possibility of amplifying non-target bacterial and eukaryotic genes. Indeed, the specificity of the *cpn*60-based method we describe was low (43%), which suggests a high rate of false positives by this method. However, the sequences of these amplicons were identical or nearly identical to ‘*Ca.*Phytoplasma’ spp., demonstrating that rather than being false positive by the *cpn*60-targeted assay, these samples were false negatives by the P1/Tint assay. In fact, the sensitivity of the 16S-23S-targeted PCR assay using the *cpn*60-targeted assay as a gold standard was only 0.745 (95% CI 0.069), suggesting that the *cpn*60-targeted PCR assay we have described is more sensitive than the 16S-23S-targeted assay.

The molecular phylogeny of ‘*Ca.*Phytoplasma’ spp. that is described using *cpn*60 UT sequences is congruent with what has been described based on 16S rRNA-encoding sequences [7], [32], with three major clades identified. Group 1 is a basal clade that includes sequences derived from ‘*Ca.*P. *asteris*’ (AY; 16SrI) and ‘*Ca.* P. *solani*’ (Bois noir; 16SrXII). The second clade consists of strains identified as Apple Proliferation, Pear Decline, and European Stone Fruit Yellows (16SrX). The third clade, which is by far the most divergent both by 16S rRNA gene sequences and *cpn*60 UT sequences, consisted of a large number of 16Sr groupings. We amplified the *cpn*60 UT from three members of this clade (‘*Ca.*P. *fraxini*’, ‘*Ca.*P. *ulmi*’, ‘*Ca.*P. *phoenicium*’) and, despite the fact that we were unable to source DNA from an isolate of PnWB phytoplasma (16SrII), we would expect to be able to amplify its *cpn*60 UT since its sequence was used as the basis for the primer design in this study.

While these sequences represent a diverse sampling of the breadth of phytoplasma taxonomy, we were not able to access template DNA from all described ‘*Ca.*Phytoplasma’ spp. (or all representative 16Sr groups). For this reason it is not certain that the primer sets we describe will successfully amplify *cpn*60 from all known ‘*Ca.*Phytoplasma’ spp. This fact highlights one of the most important limitations of the phytoplasma *cpn*60 amplification strategy: genome sequencing has revealed that certain ‘*Ca.*Phytoplasma’ spp. belonging to 16SrIII (including X-disease) lack the gene encoding Cpn60 [45], as has been noted in certain Mollicutes [46]. This does not necessarily mean, however that all phytoplasma in the 16SrIII group lack *cpn*60. Moreover, while a strength of the strategy we describe is the diversity of the sequences amplified, this very diversity may limit the ability to access novel *cpn*60 sequences from highly divergent ‘*Ca.*Phytoplasma’ spp. DNA extracts from samples of unknown status that test negative by the *cpn*60 PCR assay may therefore be negative, may contain PCR inhibitory compounds, may contain a phytoplasma *cpn*60 that is not recognized by the primers, or may contain DNA from a strain of phytoplasma that lacks *cpn*60. The first two possibilities can be countered using an internal amplification control [47], but the latter two possibilities mean that unknown samples that test negative by *cpn*60 PCR should be screened using 16S–23S or other amplification strategies. It is also likely that the *cpn*60-targeted PCR primers described here can be changed to improve their breadth of detection as new full-length phytoplasma *cpn*60 sequences accumulate from genomic sequencing efforts, which is quickly becoming the standard for bacterial species delineation [48]–[50]. Genomic sequencing of ‘*Ca.*Phytoplasma’ spp. will also provide a more comprehensive description of species that may lack the gene encoding Cpn60.

The use of *cpn*60 as a diagnostic target for ‘*Ca.*Phytoplasma’ spp. presents advantages regarding strain resolution compared to previously described 16S-23S-targeted PCR assays [14], [15]. The novel *cpn*60-based molecular diagnostic assays we have developed provide a set of tools to answer various questions regarding the presence of phytoplasma DNA in a sample. First, phytoplasma PCR followed by gel electrophoresis can sensitively determine if the sample contains DNA from a strain of phytoplasma that encodes Cpn60 in its genome, providing a binomial (positive/negative) result. Second, hybridization to oligonucleotide-coupled fluorescent microspheres provides similar binomial data but also rapidly returns information regarding the specific strain of phytoplasma that is present in the sample without the need for gel electrophoresis or amplicon sequencing. Application of these assays revealed a differential pattern of phytoplasma strain infection between *Camelina sativa* and *Brassica napus* that was difficult or impossible to identify using 16S–23S rRNA-encoding sequences. The identification of both AY-OY-M and AY-WB in *B. napus* plants but only the former strain in *C. sativa* plants from the same geographic location (and presumably the same potential pool of vectors) suggests that different vectors might have infected *B. napus* and *C. sativa*. In Saskatchewan, the aster leafhopper (*Macrosteles quadrilineatus*) is considered to be the main AY vector in oilseed crops [51], but other known AY vectors have been identified in and around those crops, sometimes in high numbers (Soroka et al, Can. Entomol., in press). Alternatively, *C. sativa* may have been infected with vectors carrying both subtypes but was only able to support the replication of one of the subtypes of phytoplasma. Multiple infections have been described in insect vectors [1] and in perennial and annual plants [51], [52]. Other explanations of this differential infection pattern such as vector feeding behavior or attraction/repellence from the host plants are also possible, and the biological significance of this observation is presently unknown. However, the detection of multiple infections is of consequence regarding the development of tools (whether molecular or bioassays) to screen for phytoplasma resistance in oilseeds. The *cpn*60-based diagnostic tools we have described provide a means to address this and other questions related to phytoplasma strain distribution and plant and insect vector susceptibility. The availability of new *cpn*60 sequence data for phytoplasma also facilitates the development of other molecular diagnostic assays, such as loop-mediated isothermal DNA amplification [30] and quantitative PCR. These *cpn60*-based diagnostic tools will provide an important complement to existing 16S–23S, *tuf*, *secY*, *rpoB*, and other molecular diagnostic assays for phytoplasma.

## Supporting Information

S1 Fig Sequence alignment of *cpn60*-targeted PCR primer hybridization sites. Sequences are identified by their cpnDB ID numbers (cpndb.ca), or by their sample of origin; primer hybridization sites for plant samples were determined from sequences generated by primer pair AY-groELF-H280p (see text for details). These sites are immediately upstream (A) and downstream (B) of the universal target sequences described in this manuscript. The downstream sequences are reverse-complemented relative to the coding region of the *cpn60* gene for clarity. The sequences of the cpn60-targeted amplification primers are shown below each alignment. Sequence logos demonstrating base distributions at each site were generated using the tool provided at http://weblogo.berkeley.edu/logo.cgi.
(PNG)

S2 Fig **Evidence of a mixed infection in** ***B. napus*** **sample BN28T-94.** The PCR product generated from H279p/H280p was directly sequenced using the amplification primers. The indicated position was called as an ‘A’ but a clear ‘C’ signal can be seen on both strands. Similar results were observed at 11/11 sites of difference between these two sequences (S4 Table).
(PNG)

S3 Fig ***cpn*** **60 UT sequences identified in samples collected from infected** ***B. napus*** **and** ***C. sativa*** **plants.** Two sequences were identified in the *B. napus* samples that were similar or identical to both AY-OY-M and AY-WB while the *C. sativa* samples displayed evidence of only a single strain of phytoplasma, AY-OY-M. The sequences of AY-OY-M and AY-WB were 98% identical.
(PNG)

S4 Fig 16S-23S-encoding sequences identified in the same pool of infected *B. napus* and *C. sativa* samples.
(PNG)

S1 Table Phytoplasma strain sources.
(XLSX)

S2 Table Oligonucleotide sequences and amplification conditions.
(XLSX)

S3 Table *cpn60*-based PCR/sequencing and fluorescent microsphere assay results on infected and uninfected *B. napus* plants.
(XLSX)

S4 Table Evidence of mixed infection of AY-OY-M and AY-WB (Ca. *P. asteris*) in *B. napus* sample BN28T-94 revealed by fluorescent microsphere assay.
(XLSX)

S5 Table Determination of Phytoplasma strains infecting *B. napus* and *C. sativa* plants grown at the AAFC Experimental Research Farm in Saskatoon, Saskatchewan, Canada, in 2012.
(XLSX)
