# Supplementary figures and images for: Molecular Diagnostic Tools for Detection and Differentiation of Phytoplasmas Based on Chaperonin-60 Reveal Differences in Host Plant Infection Patterns
Source: PLoS One. 2014 Dec 31;9(12):e116039. doi: 10.1371/journal.pone.0116039 (PMC4281212; doi:10.1371/journal.pone.0116039)

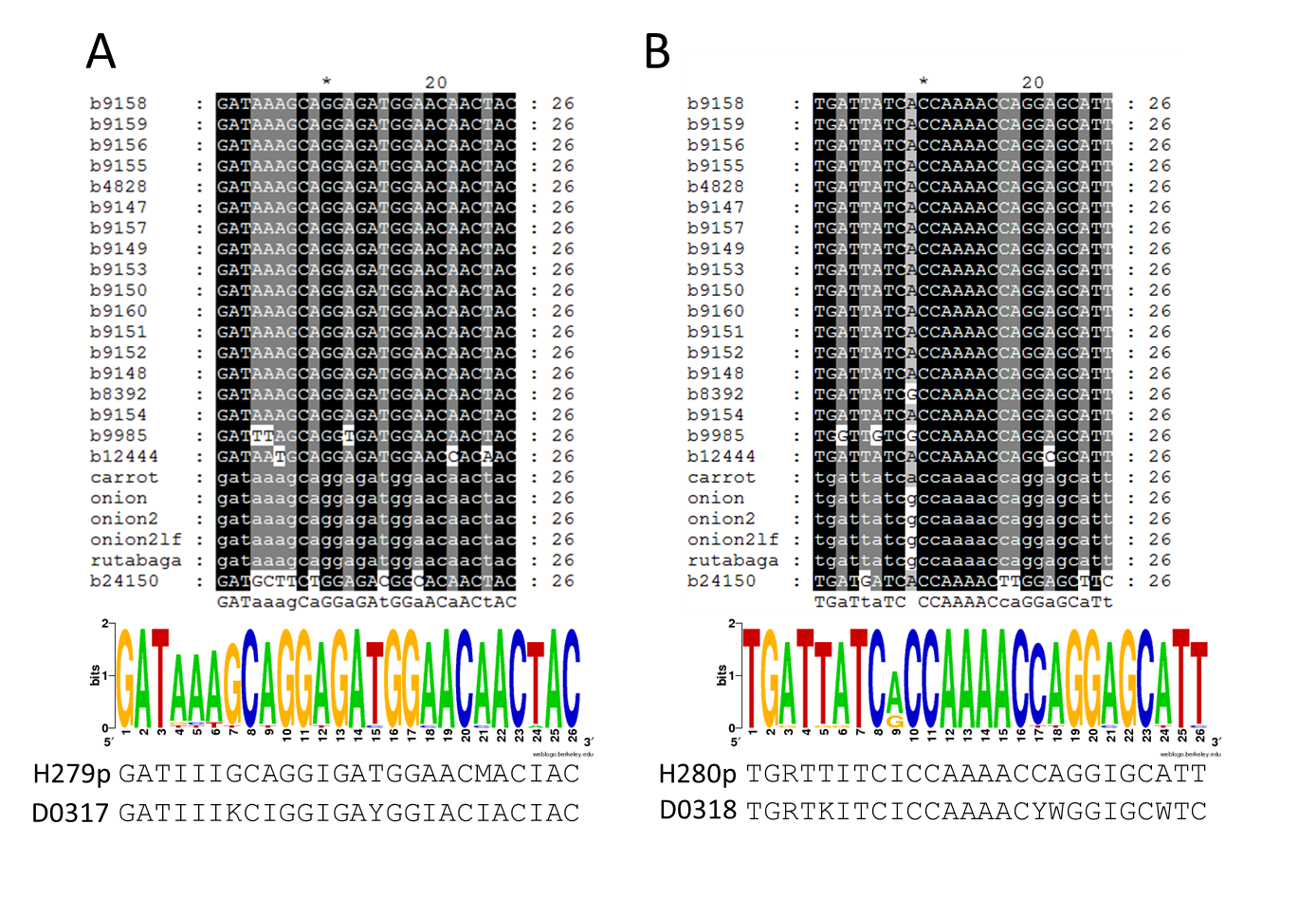

Supplement: S1 Fig — Sequence alignment of cpn60-targeted PCR primer hybridization sites. Sequences are identified by their cpnDB ID numbers (cpndb.ca), or by their sample of origin; primer hybridization sites for plant samples were determined from sequences generated by primer pair AY-groELF-H280p (see text for details). These sites are immediately upstream (A) and downstream (B) of the universal target sequences described in this manuscript. The downstream sequences are reverse-complemented relative to the coding region of the cpn60 gene for clarity. The sequences of the cpn60-targeted amplification primers are shown below each alignment. Sequence logos demonstrating base distributions at each site were generated using the tool provided at http://weblogo.berkeley.edu/logo.cgi. (PNG) [file pone.0116039.s001.png]

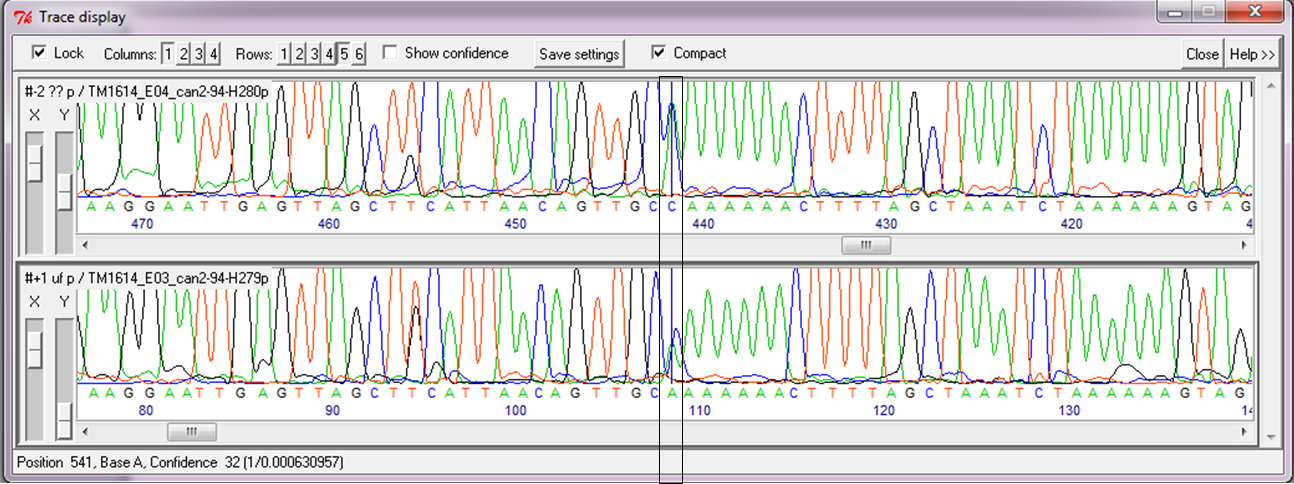

Supplement: S2 Fig — Evidence of a mixed infection in B. napus sample BN28T-94. The PCR product generated from H279p/H280p was directly sequenced using the amplification primers. The indicated position was called as an ‘A’ but a clear ‘C’ signal can be seen on both strands. Similar results were observed at 11/11 sites of difference between these two sequences (S4 Table). (PNG) [file pone.0116039.s002.png]

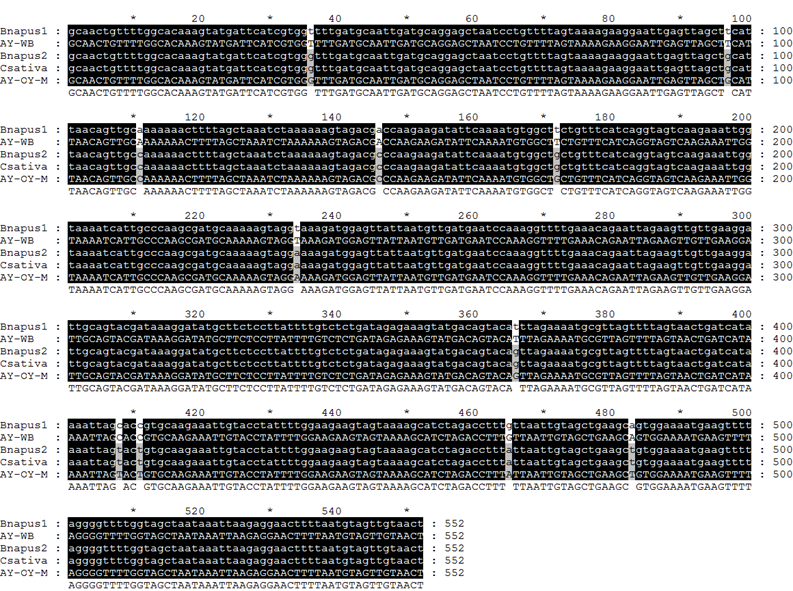

Supplement: S3 Fig — cpn 60 UT sequences identified in samples collected from infected B. napus and C. sativa plants. Two sequences were identified in the B. napus samples that were similar or identical to both AY-OY-M and AY-WB while the C. sativa samples displayed evidence of only a single strain of phytoplasma, AY-OY-M. The sequences of AY-OY-M and AY-WB were 98% identical. (PNG) [file pone.0116039.s003.png]

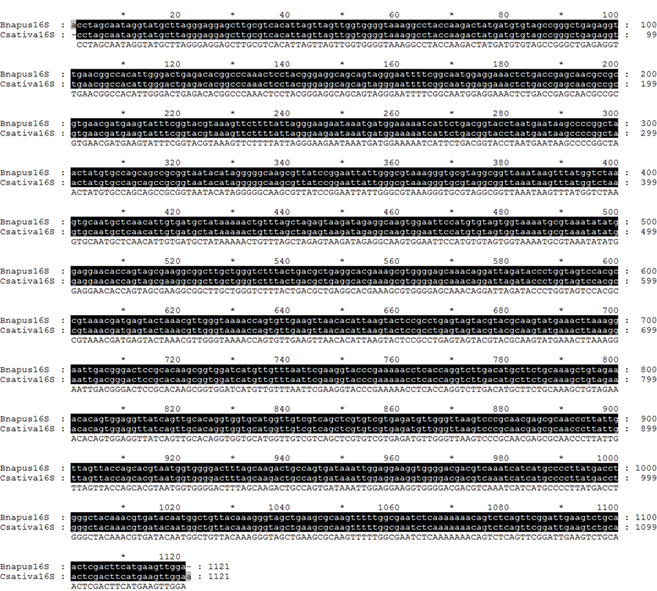

Supplement: S4 Fig — 16S-23S-encoding sequences identified in the same pool of infected B. napus and C. sativa samples. (PNG) [file pone.0116039.s004.png]
